# Habitat selection during dispersal reduces the energetic cost of transport when making large displacements

**DOI:** 10.1098/rspb.2025.1442

**Published:** 2025-11-26

**Authors:** Tullio de Boer, Kennedy Sikenykeny, Brendah Nyaguthii, Damien R. Farine, James A. Klarevas-Irby

**Affiliations:** ^1^ Department of Evolutionary Biology and Environmental Studies, University of Zurich, Zurich, Switzerland; ^2^ Mpala Research Center, Nanyuki, Kenya; ^3^ Faculty of Veterinary Medicine, University of Nairobi College of Agriculture and Veterinary Sciences, Nairobi, Kenya; ^4^ Division of Ecology and Evolution, Australian National University, Canberra, Australia; ^5^ Department of Ornithology, National Museums of Kenya, Nairobi, Kenya; ^6^ Department of Collective Behaviour, Max Planck Institute of Animal Behavior, Radolfzell, Germany; ^7^ Department of Migration, Max Planck Institute of Animal Behavior, Radolfzell, Germany

**Keywords:** dispersal, habitat selection, step selection, vulturine guineafowl, movement ecology, energetic efficiency

## Abstract

Dispersal is energetically costly. However, there is now growing evidence that dispersing animals can express distinct movement strategies that allow them to mitigate most of the energetic costs of displacing over large distances. While to date we know that these strategies involve changes in how dispersing animals move, it is unclear whether these changes in movement are facilitated by other components of behaviour—namely changes in habitat selection. Here, we collected high-resolution GPS tracking data in terrestrially dispersing vulturine guineafowl (*Acryllium vulturinum*) to test the hypothesis that dispersing animals should select for habitats that facilitate more energetically efficient movements during dispersal. Using step selection analyses, we find that actively dispersing individuals exhibit increased positive selection for open habitats, especially roads. We then use models of the energetic costs of movement to show that moving along roads facilitates straighter, faster movements and results in a more than 33% reduction in the energetic cost of transport relative to other habitat types. Our results confirm that fine-scale differences in habitat selection expressed by dispersers facilitate more energetically efficient movement, expanding our understanding of how animals exhibit adaptive movement strategies across different axes of decision-making (e.g. where and how to move) to overcome ecological challenges.

## 1. Introduction

Dispersal is a complex and often costly process. Many factors drive individuals to leave their natal habitats [1–3], including avoiding inbreeding or competing with kin [4,5], gaining new mating opportunities [6], or escaping suboptimal environmental conditions [7,8]. The costs associated with dispersing are illustrated by the striking adaptations that organisms have evolved to enable long-distance movements, such as wings [9–11], floating or rafting behaviours [12], and mechanisms for seed movement [13,14]. In some species (e.g. many Hymenoptera [15,16]), traits that facilitate increased movement are expressed exclusively in the context of dispersal, highlighting that dispersal is a distinct life stage with its own trade-offs and adaptations. While many animals cannot express extensive morphological plasticity across life stages, there is nonetheless substantial scope for evolving behavioural strategies to overcome the challenges individuals face when dispersing.

Dispersing typically requires individuals to make much larger displacements than they do throughout the rest of their lives [17]. It has long been assumed that such displacements necessarily invoke greater energetic expenditure [17,18]. Logically, moving several times further than normal [19,20] must consume more energy. However, studies from several terrestrial species (including bears (*Ursus arctos*) [21]*, lions (Pantera leo)* [22]*,* vulturine guineafowl *(Acryllium vulturinum)* [20] *and elk (Cervus canadensis)* [23] among others [24,25]) have revealed that animals can employ key behaviours during dispersal to mitigate these costs. Specifically, actively dispersing (transient) individuals increase the speed and straightness of their movements, thereby gaining greater energetic efficiency. This is because the nonlinear relationship between movement speed and energetic expenditure [26] means that while moving faster uses more energy per unit of time, the corresponding time savings result in less energy used to cover a given distance relative to slower movement (i.e. greater energetic efficiency over space). Making straighter movements also increases efficiency by covering less overall distance than when making tortuous movements, and because turns are also costly [27]. While the relative energetic impact of these changes in behaviour are rarely quantified (although see [25]), recent work in vulturine guineafowl found that efficiency-driven behavioural strategies can allow individuals to achieve much larger displacements with minimal increases in energy use relative to non-dispersal movements [20]*.*

While increased movement speed and path directedness can result from behavioural changes, habitat characteristics, such as vegetation density, should also determine the ability for individuals to express these movement characteristics [28,29]. Dispersing African wild dogs (*Lycaon pictus*) typically make larger or more directed movements in open (e.g. grassland) versus closed (e.g. woodland) habitats [30], a pattern mirrored in several other species (e.g. elk *C. canadensis* [23] *and red squirrels Sciurus vulgaris* [31]). During foraging movements, olive baboons *(Papio anubis)* preferentially move along roads in the morning and evenings, when commuting between sleeping and foraging sites [32]. However, like many aspects of the transient phase of dispersal [33], habitat selection during dispersal (relative to movements within settled home ranges) have been overlooked as a means to mitigate movement costs. In particular, animals faced with needing to move large distances should be expected to selectively use habitats that facilitate faster, straighter (and thus more efficient) movements.

Animals frequently express flexible habitat use according to their current needs. For example, many animals switch between open foraging areas and shade or cover to maintain thermal homeostasis [34] or avoid exposure to predators [35]. When making large dispersal movements, we expect that transient animals should also adjust their habitat use by disproportionately selecting those that maximize energy-efficient movements. Specifically, they should select more open habitats as these have fewer barriers and facilitate navigation owing to greater visibility. However, open habitats may also provide benefits in terms of increasing the likelihood of detecting conspecifics [36], whether to facilitate detecting groups [37] or to avoid aggression from territory owners [38]. Thus, while selectivity for open habitats during dispersal has been observed in several species, it has yet to be confirmed whether such preferences translate to increased energetic efficiency.

Here we use high-resolution GPS tracking in a wild population of vulturine guineafowl to assess (i) the habitat selection behaviours of dispersing individuals, and (ii) quantify whether selected habitats reflect a lower cost of transport (CoT; a measure of efficiency capturing the energy consumed to move a given distance). Vulturine guineafowl are almost exclusively terrestrial and live in groups ranging from 13 to 65 individuals [39]. Dispersing individuals can walk up to 15 km per day, mostly alone [20], traversing a mixture of savannah scrub, open grassy areas and roads—all habitat features present in their natal home range. Guineafowl are also a model system for physiology, with measured energetic expenditure when walking at different speeds and inclines [40]. Previous work combining physiological models with second-by-second movement data in the wild has revealed that dispersing individuals consume only 4.1% more energy for movement, despite moving 33.8% further than when not dispersing [20]. Dispersal studies typically compare the dispersal period to the behaviours of the same individuals prior to dispersing (e.g. [41,42]). However, in many species, including buzzards (*Buteo buteo*, [43]) and wolves (*Canis lupus*, [44]), the timing of dispersal reflects changing in ecological conditions [45,46]. In the vulturine guineafowl, dispersal occurs after intense rainfall [47], which also alters habitat conditions. Because vulturine guineafowl remain in temporally stable groups when not dispersing [48], we can directly contrast the habitat selection behaviours that individuals express during dispersal to those that they would have expressed (as part of their group) if they were not dispersing, providing a direct test of the effects of habitat selection on energetic efficiency. Further, dispersers alternate between days of large displacement and local prospecting [47], allowing us to test whether changes in habitat selection correspond to active dispersal (i.e. making large displacements).

## 2. Material and methods

### (a) Overview

**Figure 1.**
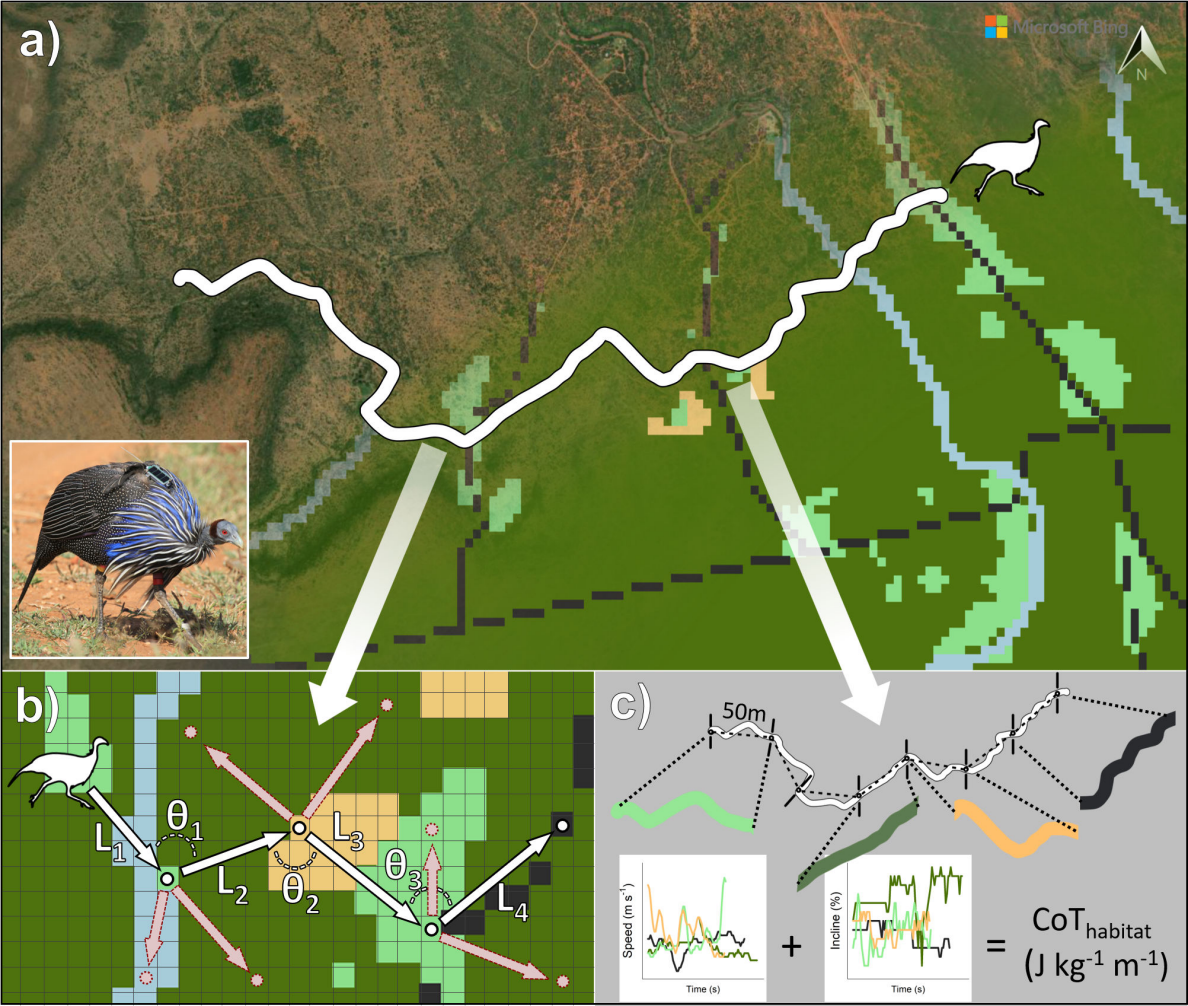
Overview of study methods and analyses. (a) GPS tracks (white) of wild vulturine guineafowl movement (GPS tagged guineafowl, inset, photo by D.R.F.) in Laikipia, Kenya matched to habitat rasters including roads (black dashed lines), acacia scrub (dark green) and more open habitats like glades (light green) and bare soil (orange). (b) Step-selection analysis using observed steps from the GPS track taken over 5 min intervals (white arrows and points) and alternative steps (dashed red arrows and points, generated based on observed step length (L) and turning angle (θ) distributions). (c) Cost of transport (CoT) for each habitat type—based on movement speed, incline and path straightness—is calculated by extracting 50 m net displacement segments and estimating the energetic CoT by inputting movement speed and incline into physiological models. Satellite imagery generated using Bing Maps, Maxar Technologies (accessed May 2025).

Our study consisted of four components (figure 1):

(i) field data collection: we collect high-resolution movement data by fitting GPS tags to subadult vulturine guineafowl and to adults from their natal group;
(ii) environmental data collection: we used remote sensing to extract features of habitats used by both residents and dispersers;
(iii) step-selection analysis (SSA; figure 1b): we used SSA [49] to quantify habitat selection across three movement categories: large active dispersal movements, local movements during dispersal and non-dispersal movements by resident adults (date-matched to dispersers’ movements); and
(iv) estimating energetic efficiency across habitats (figure 1c): we combined physiological models with high-resolution (1 Hz) GPS data to quantify the average energetic efficiency of movement (the net CoT) when moving through each habitat.

We performed all analyses in R version 4.3.1 [50].

### (b) Study system

This study was conducted at the Mpala Research Centre in Laikipia county, central Kenya (0.29120 N, 36.898670 E), which covers an area of roughly 200 km^2^ and is part of the Greater Ewaso Ecosystem [51]. Because vulturine guineafowl disperse outside of Mpala, our study extends beyond these boundaries to cover approximately 769 km^2^. This landscape contains three major habitat types. More elevated areas contain a clay vertisol commonly known as ‘black cotton’ soil [52] and are dominated by whistling thorn (*Acacia drepanolobium*). This habitat is largely avoided by vulturine guineafowl. The lower part of the escarpment, with friable ‘red soils’, contains a mixture of acacia scrub and open glades (grassy areas) as well as riverine habitats with fever trees (*Vachellia xanthophloea*) [53]. Vulturine guineafowl predominantly reside on these red soils. All major habitat types are crossed by numerous unpaved roads and smaller vehicular tracks used for research and management.

Our study on vulturine guineafowl has been running since 2016 [39]. The present study, however, relies on a focused period (7 September to 25 November 2019) during which we fitted GPS tags (see below) to female vulturine guineafowl that were still in their natal group. Dispersal is exclusive to females, typically solitary and occurs around 2 years of age, when individuals are physically mature [20]. Dispersing guineafowl do not form new groups but disperse between existing groups [47], occasionally failing to settle into a new group (because of social resistance, *sensu* [54]) and returning to their natal group for several months before trying again. There are typically two dispersal seasons per year, corresponding to the major and minor wet seasons, with our study corresponding to the minor wet season of 2019.

We also used data from non-dispersing resident members of the natal groups fitted with GPS tags during the same time period. This allows us to compare movements under identical environmental conditions and robustly quantify differences in the types of habitats selected. Notably, group movements in this species are democratic ([55]; any given individual can initiate movement in a preferred direction), facilitating comparisons of habitat selection behaviours between dispersing individuals and residents.

### (c) Field data collection

Birds were captured using baited walk-in traps and marked with a uniquely numbered stainless-steel ring and a distinct combination of four plastic colour bands on their legs. We selected 10% of adult group members to be fitted with a GPS tag (15 g Bird Solar, e-obs GmbH) using a backpack-type harness (see [39] for more details). For this study, we also fitted 18 dispersal-age females (from four social groups) with GPS tags to complement 13 GPS-tagged resident adults (nine males, four females). The combined weight of backpacks, tags and leg markings is less than 3% of birds’ body weight.

We programmed tags to run from 6.00 to 19.00 local time (corresponding to daylight hours). Date, time and location were recorded at two resolutions: continuous high-resolution (i.e. 1 Hz) when tag batteries had a high level of charge (up to 4.5 h continuous recording, approximately every second to third day), and lower resolution (a 10 s burst of 10 fixes every 5 min) when battery levels fell below the high-resolution threshold. He *et al.* [56] provide details on the strategy behind this sampling design. Data were remotely downloaded over a secure VHF connection at least every other night. For transient individuals, we downloaded data every morning and evening to opportunistically detect active dispersers and obtain a movement bearing to facilitate future relocation. Some birds ultimately dispersed beyond areas that we could access (three such individuals were eventually re-encountered several months later and their accumulated data recovered). GPS data were uploaded to the Movebank repository (https://www.movebank.org) for remote storage and prepared for analysis in R through the move package [57].

#### (i) Dispersal

We defined three stages of dispersal (departure, transience and settlement) based on movement patterns. Birds were considered to have started dispersing on the first day that their GPS tracks diverged from the resident adults in their natal group. Dispersers were subsequently considered to have settled in a new area after using the same roosting site for 14 days (back-dated to the first date after they entered the new roost). We observed no further large displacements by a given individual later than the 14 day cut-off and field observations confirmed that dispersers were part of a new group. All GPS data between the departure and settlement dates for each individual were then treated as transience. In 11 cases, we were unable to record settlement. In six of these cases, the dispersing individual moved beyond where we could download data. In one case, the GPS tag fell off owing to a failure of the harness materials. In three cases, the dispersing individual failed to settle and returned to the natal group after several weeks. Finally, one individual was predated during transience. In these cases, we used transience data up to the available end point. As we focused on habitat selection behaviours by transient birds, all pre-departure and post-settlement GPS data were excluded from our analyses. Instead, for each disperser, we included date-matched GPS data from all available resident adults of each dispersers’ natal group to capture how they would have moved if they were not dispersing.

Dispersal movements during transience are not uniform in vulturine guineafowl [47,58], but have two distinct daily patterns. The first involves making large roost-to-roost displacements, corresponding to days of active dispersal. The second involves movements that take place over much smaller, local scales (typically without changing roosts from morning to evening). Field observations suggest that local movements occur when dispersers temporarily join new groups, whereas large displacements are typically made alone. Both movement types are interspersed and can occur for one or more days. We thus categorize each transience day for each disperser as either active dispersal or local prospecting. An individual was considered to have engaged in large, active dispersal movements if the distance between consecutive days’ roosts exceeded 1500 m, or if the roost-to-roost distance was greater than 1200 m and the ratio of this distance to their total daily track length (i.e. a straightness index [59]) was greater than 0.3 [47]. Sub-categorizing the movements of transient individuals in this way allowed us to determine whether habitat selection patterns during transience correspond to making large movements. See the electronic supplementary material for a description of the total GPS dataset.

### (d) Environmental data collection

#### (i) Generating habitat raster layers

**Figure 2.**
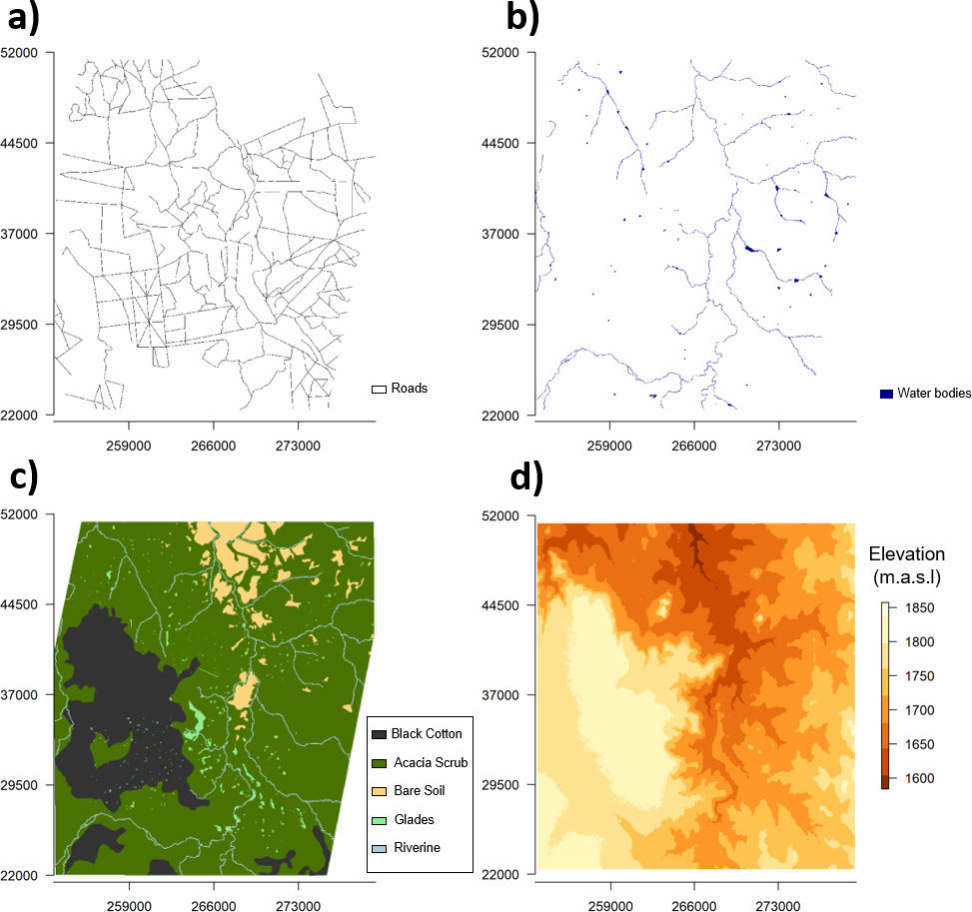
Raster layers of habitat covariates across our study area. We captured the presence of roads (a) and waterbodies (b), classified cover type (c) and obtained elevation data in metres above sea level (d). Axes show Universal Transverse Mercator (UTM) coordinates.

To measure and define the overall habitat composition of our study area, we downloaded satellite imagery from Planet Explorer by Planet Labs [60]. We created a bounding box encompassing all of our GPS data to build the base geometry for the search and download of satellite imagery. We selected PlanetScope Scene imagery (hereafter, ‘satellite imagery’) from 3 November 2019 (resolution: 3 m, area coverage: 94%), as it best satisfied our criteria (maximum area coverage, no cloud coverage) within the period when we recorded the majority of our recorded dispersal events. Satellite imagery was then prepared and processed for analysis in ArcGIS’ ArcMap 10.6.1. [61]. Satellite images were georeferenced and projected to WGS84 UTM Zone 37N (EPSG: 32637). We then manually extracted three raster layers (figure 2) capturing roads, water bodies and habitat type (the latter being classified into five types).

Roads and water body layers were both defined as either present (1) or absent (0) for each pixel. As no road dataset was available for our study area, we manually marked roads as polylines. All roads are rural gravel roads, with no heavy-use roads present in the study area. With our spatial resolution of 3 m, it was not always easy to distinguish between smaller or older/abandoned roads and animal paths. Thus, we opted for including roads that were distinguishable through visual inspection of our satellite data and Google Earth™. We defined roads as present using polygons extending 15 m to either side of the centre of each polyline.

For water bodies, we followed the same approach as for roads to obtain polylines for rivers (buffering 15 m on each side). Ponds, dams and small lakes were drawn as polygons and merged with the river features. Water pixels could be distinguished from other landscape features based on their reflection in false colour infrared or in the normalized difference vegetation index (NDVI). The main component for the water bodies layer in our study area is the Ewaso Ng’iro river. There are also several ponds, small lakes and dams; the largest (0.3 km^2^) being a dam on the neighbouring Ol Jogi reserve.

We categorized habitats into five cover types using the false colour infrared band combination and comparison to Google Earth scenes with higher spatial resolution and true colour. Black cotton soils, owing to their dark colour, were easily detectable as dark tones in the false colour infrared spectral combination. Glades (open grassy areas) appeared in vivid red in the same band combination owing to the higher chlorophyll content found in the plant species on open glades (green reflectance shows as red in false colour infrared). Bare soil represents sandy patches with little to no vegetation and was distinguishable as white pixels. The predominant habitat cover was dense acacia scrub, which presented as a dark reddish colour owing to its lower chlorophyll content compared to glades. Finally, we classified a 30 m buffer zone along marked rivers as riverine habitat.

#### (ii) Generating the topography raster layer

To quantify topography, we used a 30 m resolution digital elevation model (DEM) from the Open Data Site of the Regional Centre for Mapping of Resource for Development (opendata.rcmrd.org). We extracted the slope and aspect of inclined surfaces, as well as a terrain ruggedness index (TRI) that quantifies average topographic heterogeneity [62]. TRI values were calculated by taking the DEM values from a centre cell and the differences to its surrounding eight neighbour cells (we ground-truthed the DEM by measuring elevation at 24 locations in the study area). The differing values to each of the eight neighbour cells were then squared, summed up and subsequently square rooted [62], which quantifies the topographical heterogeneity in a given area with higher values corresponding to more rugged or uneven terrain.

### (e) Step-selection analysis

We used the ‘amt’ package [63] in R to conduct an SSA to assess how landscape features influence where vulturine guineafowl move according to different stages. This type of analysis uses positional data to capture movement steps. For each observed step (i.e. two consecutive relocations in our GPS data) we generated 20 alternative steps [49] that represent plausible alternative locations in which the individual could have moved (given the properties of its movements). This produced a dataset allowing us to compare the environmental features in observed and plausible alternative steps to identify which were over-represented in the observed steps.

#### (i) Temporal discretization of observed steps

To maximize the continuity and coverage of our data, we opted to divide our data into 5 min steps (combining continuous, 5 min data with sub-sampled 1 Hz data). After calculating step lengths (net displacements) for each 5 min interval, we removed all steps shorter than 10 m to focus on where an animal moved rather than on the timing of movement [32]. In this way, the resulting model is less affected by periods of little or no movement. We then marked the next time point as the start of a new step, and repeated the process for the rest of the track. From an initial dataset of 132 959 five minute GPS intervals, we obtained a final dataset with 61 435 observed steps greater than our 10 m threshold.

#### (ii) Generating alternate steps

We recorded the true step length (in metres) and the turning angle between consecutive steps (in radians). The distributions of these values (electronic supplementary material, figure S1) were subsequently used to generate the alternative steps for our SSA. For each individual and each category of movement (i.e. active dispersal or local movements by transient birds, and movements of residents), we fitted a gamma distribution to the observed step lengths and a von Mises distribution to observed turning angles [64]. For each observed step, we then generated 20 alternative steps based on the individual-and-category-level distributions of step lengths and turning angles.

#### (iii) Linking steps to habitat features

Using the ‘raster’ package [65] in R, we combined our dataset of observed and alternate steps with our environmental covariates. We first imported our prepared environmental raster layers (i.e. presence/absence of waterbodies and roads, habitat cover type and a terrain ruggedness index) and then projected our step locations onto them. For each step, we extracted the relevant environmental covariates from the raster cell which contained the endpoint of that step.

#### (iv) Fitting a step selection function

Using ‘amt’, we fitted a wrapper function of a conditional (fixed-effects) logistic regression for matched case–control (observed versus alternative step) data with the R package ‘survival’ [66]. We fitted the conditional logistic regression using the same routine as a Cox proportional hazards model [63]. Each observed step and its matching 20 alternatives formed a stratum. We fitted two models, which produce comparable selection coefficient estimates, but which evaluate significance based on differing criteria (see the electronic supplementary material, tables S1 and S2 for detailed model formulas). The first model assessed whether selection behaviours exhibited within each movement stage (i.e. resident, local-transient or actively dispersing transient) were significantly positive or negative (representing selection or avoidance, respectively). This first model included an interaction term between each environmental covariate—water bodies, roads, cover type (with acacia scrub as the reference level) and terrain ruggedness—and a three-level categorical factor representing movement stages (residents were set as the reference level). The second model assessed whether dispersing birds exhibited selection behaviours that differed significantly from those of non-dispersing residents (i.e. regardless of whether the overall selection strength was positive or negative). This second model featured a standalone term for each of our environmental covariates as main effects, in addition to the interaction term for each environmental covariate with movement. We also constructed a second pair of supplementary models (see the electronic supplementary material, tables S3 and S4) with only two levels of interaction (transient and resident, irrespective of the type of daily movement during dispersal) to enable comparisons between the present study and other systems where analyses are more typically divided based on whether or not individuals are dispersing. Models estimate habitat selection as the relative strength of selection (RSS) for a given habitat feature, corresponding to the probability of selecting for a location that corresponds to a given feature relative to the reference level (assuming both options are equally available).

Non-dispersing residents made no observed steps into areas with black cotton or bare soils (electronic supplementary material, table S5), and initial models showed no strong evidence for selection or avoidance of these habitats by dispersers. Thus, we made the post hoc decision to remove all steps (observed and alternate) in black cotton and bare soils, and re-fitted all of our step selection models without them. This avoided spurious estimates of selection where residents were used as the reference category for movement stage but where they were not represented in the data for these habitat types.

### (f) Estimating energetic efficiency across habitats

We conducted a separate analysis to estimate the energetic efficiency of movement (here the energetic CoT) for each habitat and stage (i.e. non-dispersing residents, localized transient movements or transient birds making large displacements). Because most dispersing individuals moved through areas that were not used by any other GPS-tagged bird, we could only calculate efficiency metrics for observed movements (i.e. we could not infer layers of energetic landscapes). Thus, we ran this analysis independently of the SSA, generating a per-habitat measure of CoT.

#### (i) Spatial discretization of net displacements

The CoT is a measure of efficiency that describes the energy used to displace over a given distance. We obtained the CoT by first dividing our high-resolution (1 Hz) movement tracks into net displacements of a fixed size (50 m). Net displacements were extracted using the first location from each high-resolution GPS track and, for each successive time point (current location of the bird) calculating the Euclidian distance to the first location, until a threshold radius of 50 m was reached (marking the end of the displacement). We then marked the next time point as the start of a new displacement, repeating this process for the entire track. Although our threshold was set at 50 m, the true distance of a displacement often slightly exceeded this (owing to the temporally discrete sampling of our tags), and so the true distance of each net displacement was used for the calculations of the energetic CoT (i.e. per-unit-distance energy expenditure, in J kg^−1^ m^−1^).

#### (ii) Linking energetic costs to habitat features

We extracted the 50 m net displacements with start- and end-points within the same habitat or landscape feature, operating under the assumption that individuals moved through continuous habitat between the two points. If the movement took place on a road, we defined the category as ‘road’ for this analysis, independent of the habitat cover that the road crossed. For all other categories, the cover type of the movement was used as the categorical label.

For each high-resolution fix within a given net displacement, we derived the metabolic cost of movement (in ml O_2_ kg^−1^ s^−1^) at each second based on laboratory studies describing how the metabolic costs of movement vary across different controlled movement speeds (m s^−1^) and inclines in the morphologically similar, closely related helmeted guineafowl (*Numida meleagris*) [40]. Stationary guineafowl exhibit a flat rate of oxygen consumption of 19.1 ml O_2_ kg^−1^ min^−1^. When moving, oxygen consumption increases linearly with speed (v), where the slope and intercept of that relationship vary depending on the incline at which birds are moving [40]. These formulae take the form of $VO_{2}=24.0v+27.2$ on level terrain, $VO_{2}=30.7v+27.6$ at 10% incline, and $VO_{2}=47.7v+21.3$ at 20% incline [40], where V0_2_ is the per-minute volume of oxygen consumed in ml O_2_ per kilogram of body mass (ml O_2_ kg^−1^ min^−1^). While some energetic savings can be realized during downhill movements, these are often offset by increased muscle load to absorb additional impacts [67], leading to either minimal savings (e.g. [68]) or even slight increase in the CoT (e.g. in barnacle geese *Branta leucopsis* [67]). As no studies quantified the specific impact of downhill movement on guineafowl, we assumed any downhill movements followed the same cost relationship as those on level terrain.

To apply the correct cost formula to each second of data within a given displacement, we needed to determine whether an individual was actively moving. Movement was assigned according to the outputs of a 4-state hidden Markov model (constructed using the R package depmixS4 [69]) parameterized on 10 s step lengths and turning angles. We used a 4-state model because this was previously found to better capture a non-moving state than models containing fewer states [20]. When birds were moving (i.e. states 2–4), we then calculated the degree of the slope experienced based on the relationship between the individual’s movement bearing and the aspect of the underlying incline (terrain slope and aspect were derived using the DEM elevation model above), following the formula $\theta^{\prime }=tan^{-1}\left(tan\left(\theta \right)\cdot cos\left(\Delta \psi \right)\right)$ where $\theta^{`}$is the slope experienced by the individual, *θ* is the slope of the inclined plane and ∆ψ is the angular difference between the aspect of the incline and the individual’s bearing. Experienced slope values were transformed into per cent grade by the formula $PG=tan\left(\theta^{\prime }\right)*100$ where PG is the per cent grade of an incline corresponding to the slope experienced by an individual (in degrees). Experienced incline values were aligned with formulas for oxygen consumption by rounding the per cent grade at each GPS fix to the nearest tenth percentile (level terrain when the grade was less than 5%, a 10% incline when the grade was between 5% and 15%, and 20% incline when the incline was ≥ 15%). Birds never experienced grades of 30% or greater. Per-second measures of oxygen consumption were then calculated based on whether an individual was stationary or moving and, if moving, according to the speed and underlying incline. These measures were then transformed into units of energetic consumption (J kg^−1^ s^−1^) using a conversion factor of 20.1 J ml^−1^ O_2_ [40,70].

#### (iii) Estimating the energetic cost of transport within each habitat type

To calculate the CoT, we summed the per-second energetic costs within each 50 m net displacement and divided this sum by the true net displacement distance. The effect of habitat type on the energetic CoT was calculated by fitting three linear models, one for each movement stage (full details in the electronic supplementary material, table S6). An additional post hoc test showed no significant pairwise contrasts among non-road habitat variables in any of the three models, and so we only report estimated CoT values relative to roads (as modelled) here.

We note that our estimates of movement energetics are based only on speed and incline. Substrate can also impact the CoT, such as via the energy required to deform the substrate (e.g. soft sand, loose rocks, dense vegetation) or because of having to step higher up and down on uneven ground (e.g. traversing boulders). Moving on rocky terrain [71] and softer substrates [72] can substantially increase metabolic rate, and thus the CoT, independently of speed. However, there are very few data on how different substrates impact the CoT across non-human terrestrial animals [73].

## 3. Results

### (a) Dispersers differ from residents in habitat selection

**Figure 3.**
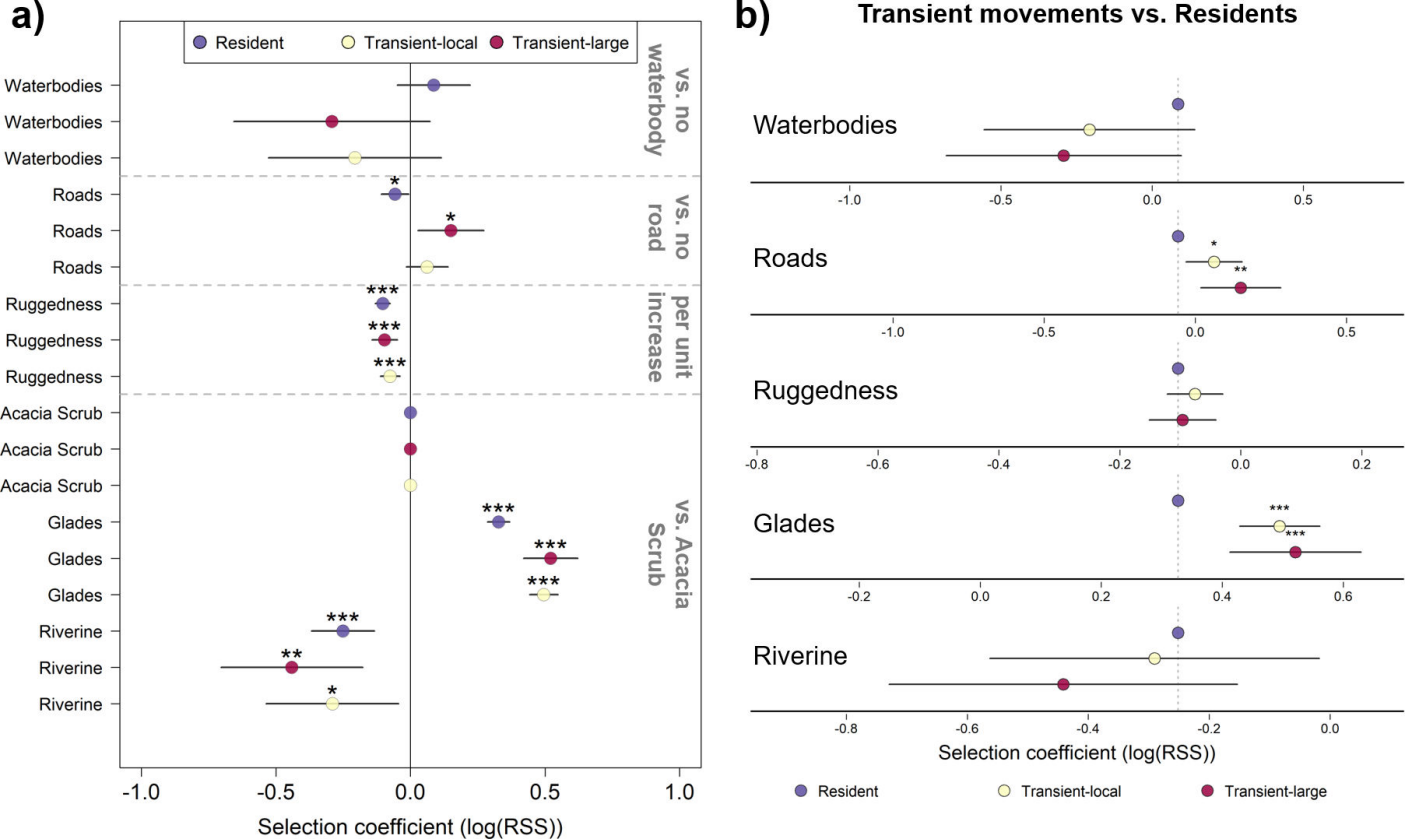
Relative selection strength (RSS) for different habitat features is distinct during dispersal. (a) Strength of selection within a given stage of movement. Points represent log-transformed estimated RSS values for a given habitat feature relative to a reference category (grey text, right; separate raster layers denoted by dashed grey lines). Black bars represent 95% confidence intervals, indicating strength of habitat selection for (where log(RSS) > 0) or avoidance of (where log(RSS) < 0) a given habitat type. Asterisks above points indicate statistical significance (***p < 0.001, **p < 0.01, *p < 0.05). Full model results and formula in the electronic supplementary material, table S1. (b) Selection strength by transient birds relative to residents. Here, statistical significance is evaluated base on relative difference to the reference category (residents—marked with purple points and vertical dashed lines). Asterisks above points (***p < 0.001, **p < 0.01, *p < 0.05) indicate where transient birds exhibited significantly stronger selection for/against a given feature than residents (i.e. regardless of whether selection differed from 0 in (a). Full model results and formula in the electronic supplementary material, table S2.

We found distinct differences in habitat selection across different stages of movement, with some contrasting patterns expressed by transient birds and residents. Non-dispersing residents expressed significant positive selection for glades (RSS = 1.382), significant avoidance of roads (RSS = 0.942), riverine cover (RSS = 0.942) and rugged terrain (RSS = 0.908) and no selection for or avoidance of waterbodies (figure 3a; see the electronic supplementary material, table S1 for full results). Actively dispersing transient birds (i.e. when making large displacements) expressed significant positive selection for roads (RSS = 1.166) and glades (RSS = 1.636), significant avoidance of riverine cover (RSS = 0.693) and rugged terrain (RSS = 0.926), and no selection for or avoidance of waterbodies (figure 3a; electronic supplementary material, table S1). During smaller, local movements, transient birds exhibited significant positive selection for glades (RSS = 1.719), significant avoidance of riverine cover (RSS = 0.724) and rugged terrain (RSS = 0.916), and neither selection nor avoidance of roads or waterbodies (figure 3a; electronic supplementary material, table S1).

When comparing habitat selection during dispersal to residents, transient birds making large displacements expressed stronger positive selection for roads (*p* = 0.002) and were also more likely to select for glades (*p* = 0.004) than residents, but did not differ significantly from residents for any other habitat features (figure 3b; see the electronic supplementary material, table S2 for full model results). Similarly, during days of local movements, transient birds again were more likely to select for roads (*p* = 0.011) and glades (*p* < 0.001) than residents, but were not different in terms of other habitat features (figure 3b; full results in the electronic supplementary material, table S2). Results of our supplementary models, which did not distinguish between transient modes, showed approximately the same results as our main models. Selection coefficients were identical for residents, dispersers selected for roads (and more strongly than residents) and glades, and avoided riverine areas and rugged terrain (similar to residents), with the one difference that dispersers in the aggregate were also significantly avoidant of waterbodies (RSS = 0.703; electronic supplementary material, figure S2 and table S3) and expressed significantly lower selection for waterbodies relative to residents (*p* = 0.012; electronic supplementary material, table S4).

### (b) Habitat selection during dispersal reflects gains in energetic efficiency

**Figure 4.**
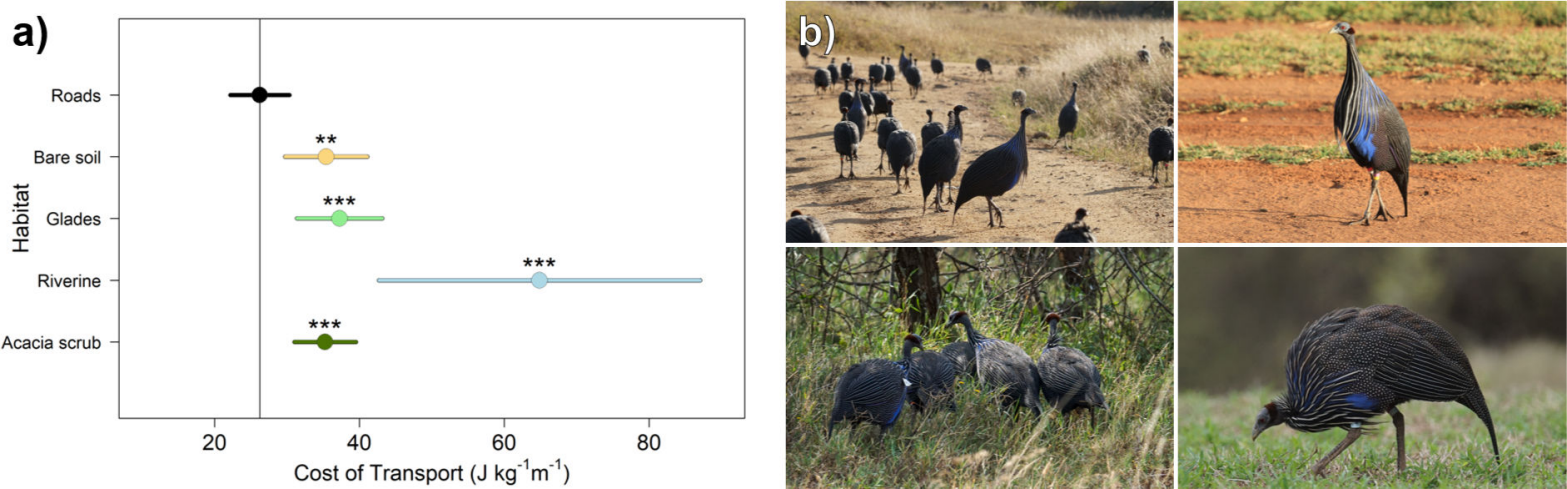
Roads facilitate significantly more efficient movements when making large dispersal movements. (a) Estimates (circles) from linear mixed-effect model of the energetic cost of transport (CoT) for actively dispersing transient birds across habitat types (coefficient ± 95% confidence intervals) using roads as the reference category (intercept marked with vertical grey line). Significant increases (i.e. higher CoT, thus less efficient movements) are marked by asterisks above points (***p < 0.001, **p < 0.01). Full results, including models of efficiency for residents and local transience movements are in the electronic supplementary material, table S6. (b) Guineafowl moving through a variety of habitats (clockwise from top left: roads, bare soils, glades and acacia scrub) with different substrate properties and vegetation densities. Photos by D.R.F. and J.A.K.-I.

Actively dispersing transient birds averaged a significantly lower energetic CoT on roads (CoT = 25.57 J kg^−1^ m^−1^, *p* < 0.001) compared with all other habitats (figure 4a; electronic supplementary material, table S6). Dispersers moving on roads experienced a 33.1% lower (*p* < 0.001) energetic CoT than when moving through the more common acacia scrub. They also experienced significantly greater efficiency on roads than on bare soil (35.9% lower CoT, *p* < 0.001) and glades (38.2% lower CoT, *p* < 0.001), with the highest CoT in riverine habitats (138% greater CoT, *p* < 0.001). Non-dispersing residents and transient birds during local movements showed no evidence for significant differences in the CoT across habitat types (electronic supplementary material, table S6).

## 4. Discussion

Our study revealed significant shifts in habitat selection by terrestrial birds during dispersal. Individuals selected for open habitats (roads and glades) when making large displacements and exhibited stronger selection for these features than non-dispersing residents. Correspondingly, we found that actively dispersing individuals experienced a significant decrease in their CoT, exhibiting the most energetically efficient movements when moving along roads. By contrast, dispersers making local movements and residents expressed only minor differences in the CoT across habitat types. Our results therefore provide support for the hypothesis that changes in habitat selection expressed by terrestrial animals during dispersal are part of a broader strategy in response to selective pressures acting on the need to achieve large displacements in an energetically efficient manner.

In many species, the factors governing habitat selection and movement are the result of trade-offs between energetic efficiency and risk [74,75]. Studies in large mammals [32,76–79] have found that individuals express preferences for linear landscape features, such as roads, when making larger displacements. However, roads can also represent areas of higher disturbance and mortality risk (i.e. owing to vehicle strikes), leading to a number of studies reporting avoidance of roads [80–82]. In our study, we found that transient, dispersing individuals expressed a strong positive selection for moving along roads, especially during days where they were actively displacing across the landscape, while non-dispersing residents avoided roads. This effect is especially strong when considering the relative rarity of roads in our study environment. That is, our models suggest that actively dispersing birds should be roughly 16.6% more likely (electronic supplementary material, table S1) to make a step onto a road when roads are equally available to areas of non-road habitat, but roads actually make up less than 3% of our total study area (electronic supplementary material, table S5). At our study site (the same area as the aforementioned baboon study [32]), most roads consist of infrequently used, private dirt tracks with easily detected low-speed traffic, meaning that the mortality risks to guineafowl is quite low (in 8 years of following a population of 600–1000 birds, we know of only two birds killed by vehicles).

By contrasting the features of different habitat types (electronic supplementary material, figure S3), we could demonstrate that the potential gains in efficiency associated with roads are because they allow birds to traverse habitat in a faster and more direct manner (electronic supplementary material, figure S4). In the areas used by guineafowl, roads are generally found on slightly steeper and more variable terrain (a small effect, but different from all other habitat features except for acacia scrub, which was slightly steeper; see the electronic supplementary material, table S7 and figure S3). This suggests that efficiency gains obtained by moving along roads are not because these coincide with areas that have less incline or are less rugged. Birds moving along roads also did not experience different inclines relative to any other habitat (electronic supplementary material, figure S4). Instead, we found that birds were faster and made straighter movements when using roads (electronic supplementary material, table S8 and figure S4). Further, while we could not account for differences in substrate consistency, we do not expect these to have large effects. This is because vulturine guineafowl largely live and move in areas with friable red soils [53], which are consistent on and off roads (figure 4b). Further, any additional impacts of substrate roughness or habitat permeability (e.g. owing to stepping through vegetation) on movement efficiency that is independent of speed should only strengthen the differences we observed between roads and scrub. Thus, our results suggest that the linearity of roads specifically allows individuals to traverse the landscape thus more efficiently.

Contrary to our expectations, other open habitats (i.e. areas with bare soil or glades) were not substantially more efficient to move through than more densely vegetated areas. Nonetheless, interesting patterns emerged in actively dispersing birds. First, the limited observations of movements on bare soils were made by transient birds (electronic supplementary material, table S5) and never by non-dispersing residents (although we could not generate a robust statistical estimate of the strength of selection), probably because bare soils generally correspond to areas with little to no suitable foraging habitat. Residents instead showed strong positive selection for glades, which are commonly used by vulturine guineafowl for foraging [39,83]. Interestingly, the single highest selection coefficient in our models (figure 3; electronic supplementary material, table S1) was for transient birds using glades (they were also significantly more likely to select for glades than residents). Given the lack of energetic savings when moving on glades, the increased selection for glades by dispersing birds may reflect other aspects of their dispersal ecology. Dispersal in vulturine guineafowl involves moving alone while trying to locate new social groups to settle into [20]. Given that the only positively selected habitats by residents was glades, it may be that dispersers are selecting for glades to locate groups. This trend is strongest during days of active dispersal movements, but was also significantly greater when making local movements relative to residents. Together, these results suggest that habitat selection by transient birds alternates between two important functions, increasing the energetic efficiency of movement on roads, and increasing opportunities to detect groups to disperse into (and potentially to increase safe foraging opportunities [84]) on glades.

In addition to changing the energetic profile, the openness of a habitat also probably shapes predation risk. In group-living species, individuals can substantially benefit from collective detection of predators (i.e. the many eyes hypothesis [84]), which play a major role in the evolutionary ecology of vulturine guineafowl. Vulturine guineafowl are large and colourful, and live in a predator-rich environment that includes a full range of terrestrial (e.g. leopard *Panthera pardus*) and aerial (e.g. martial eagle *Polemaetus bellicosus*, eastern chanting goshawk *Melierax poliopterus*) predators. Of these, vulturine guineafowl are particularly sensitive to aerial predators. In fact, the only predation event we recorded of a dispersing individual was by a martial eagle. The risk of aerial predators is reflected in dispersers moving less during the middle of the day when these predators are most active [47]. Thus, if using open habitats introduces even more risk for individuals during the active phases of dispersal, this strengthens the support for the energy efficiency hypothesis.

Our study contributes to a growing body of evidence (e.g. [30]) that habitat requirements change during dispersal. We substantially extend previous work by explicitly quantifying the energetic benefits that individuals can gain as function of both where and how they move when dispersing. However, making large displacements is not limited to natal dispersal. Many animals—especially those living in harsh environments—temporarily relocate to find resources (e.g. migration [45] or nomadism [85]), and future work may also investigate whether animals exhibit plastic habitat preferences linked to movement under different contexts. For example, migrating birds select different habitat features at stopover sites than in their non-migratory ranges [86], and several studies have shown how migratory ungulates shift between displacing and foraging modes in different habitats (e.g. [87,88]). As GPS tracking datasets also improve in coverage and sampling frequency, they should offer opportunities to investigate movement strategies more holistically. For example, time limitations for foraging in hot regions could drive strategies to increase the speed of movement, such as the ‘morning commute’ of baboons following roads to reach distant foraging areas before the day heats up [32]. Thus, our study approach could be replicated by looking at changes in habitat selection and movement characteristics at different hours of the day.

Finally, we note that our study was limited to making inferences about the selection for more energetically efficient movements in two separate analytical steps. This is because dispersers moved through areas in which we had little to no prior GPS data (unlike areas used by their natal groups), thereby limiting our ability to generate an ‘efficiency landscape’. While we could have made some inferences about the CoT for different habitats, our analysis would have been based on fitting measured CoT values in animals’ chosen steps and inferred values for alternative steps. However, future work that is focused more closely on areas with high GPS coverage (e.g. by looking at changing selection patterns over the course of the day) could use repeated observations of movements by individuals over the same areas to produce a CoT layer. This would facilitate direct tests of whether individuals are selecting for habitats with lower CoT when making larger displacements. We anticipate that such studies will provide rich grounds for gaining insights into the process of individual (or collective) decision-making by animals on the move.

## Data Availability

All data and code used to produce these results are available at [89]. Supplementary material is available online [90].
